# The challenge in the diagnosis and management of an advanced abdominal pregnancy in a resource-low setting: a case report

**DOI:** 10.1186/s13256-017-1369-1

**Published:** 2017-07-24

**Authors:** Paul N. Tolefac, Martin H. Abanda, Jacqueline Ze Minkande, Eugene Belley Priso

**Affiliations:** 1 Faculty of Medicine and Biomedical Sciences, University of Yaounde 1, Yaounde, Cameroon; 2 Service of Obstetrics and Gynaecology, Douala General Hospital, Douala, Cameroon; 3 Clinical Research Education Networking and Consultancy, Douala, Cameroon

**Keywords:** Abdominal pregnancy, Transvaginal ultrasound, βHCG, Laparotomy, Case report

## Abstract

**Background:**

Abdominal pregnancy is a rare form of ectopic pregnancy that is frequently left undiagnosed by inexperienced obstetricians and radiologists. It is associated with higher risk of maternal hemorrhage at any gestation and more at advanced gestation.

**Case presentation:**

We present the case of a 22-year-old sub-Saharan African woman, gravida 3 para 0, who was diagnosed with advanced abdominal pregnancy of 25 weeks’ gestation by a transvaginal ultrasound after the failure of two medical terminations of pregnancy in the first and second trimesters and a series of repeated obstetric ultrasounds showing intrauterine pregnancy. Laparotomy was done and her recovery was uneventful.

**Conclusions:**

The management of advanced abdominal pregnancy is more challenging as compared to earlier gestation so patients with failed medical termination of pregnancy should be critically analyzed for ectopic pregnancy as early as possible.

## Background

When implantation takes place anywhere outside the uterine cavity it is referred to as an ectopic pregnancy. Abdominal pregnancy is a rare type of ectopic pregnancy where the developing embryo implants and grows within the peritoneal cavity [1]. Advanced abdominal pregnancy (AAP) refers to a pregnancy that continues beyond 20 weeks’ gestation with a fetus living, or showing signs of having once lived and developed, in the mother’s abdominal cavity [2]. Ectopic pregnancies make up about 1–2% of all pregnancies with 95% occurring in the fallopian tube [3–5].

Abdominal pregnancies constitute about 1.4% of all ectopic pregnancies with an estimated incidence 1:10,000 live births [3, 4]. In primary AAP, there is direct implantation of the conceptus into the abdominal cavity whereas secondary AAP could be due to fimbria abortion, tubal rupture, ruptured uterus, ruptured corneal pregnancy, or as a result of intraabdominal fertilization [4, 6]. Risk factors of AAP may include uterine surgeries, dilatation and curettage, history of tubal pregnancy, and artificial insemination [7]. There are reported cases of abdominal pregnancy which were not diagnosed till surgery [8]. The clinical symptoms of an uncomplicated abdominal pregnancy described in the literature are very nonspecific, among which the most frequently encountered are persistent abdominal or suprapubic pain, missed periods, bloody vaginal discharge, and gastrointestinal symptoms like nausea and vomiting [9]. An AAP is an extremely rare condition and very few of such cases have been published during the last 10 years [3]. Therefore, there is no standard diagnosis and treatment algorithm for abdominal pregnancy. A high index of suspicion and thorough clinical and ultrasound examinations are crucial to diagnosing abdominal pregnancy [5]. Standardization of the treatment principles for advanced abdominal pregnancy, perioperative treatment options, and postoperative management measures would improve newborn survival, reduce complications, and mortality [2]. This could be done by reporting all cases observed worldwide. We present the case of a 22-year-old sub-Saharan African woman, gravida 3 para 0, who was diagnosed with advanced abdominal pregnancy (AAP) of 25 weeks’ gestation after the failure of two medical terminations of pregnancy (MTP) in the first and second trimesters and a series of repeated obstetric ultrasounds showing intrauterine pregnancy. Laparotomy was done and her recovery was uneventful.

## Case presentation

We present the case of a 22-year-old sub-Saharan African woman gravida 3 para 0, with her last menstrual period (LMP) in early August 2016. The first pregnancy was in 2011, an MTP was done at 6 weeks of gestation and there were no complications. The second pregnancy was in 2013, an MTP was done at 8 weeks of gestation and there were no complications. She presented in our service on November 30, 2016 with a positive pregnancy test, persistence of fetal movements and persistent positive signs of pregnancy after two failed MTPs for the same unwanted pregnancy. The patient reported vaginal bleeding after each failed MTP that was done by dilation and curettage. On clinical examination, her abdomen was distended with a symphysiofundal height (SFH) of 15 cm and the uterus 16 weeks size, fetal heart tones (FHT) present with a fetal heart rate of 142–148 beats/minute. Her cervix was closed, long, posterior and firm, and there was bogginess in the cul-de-sac; she was advised to do an obstetric ultrasound for localization of the pregnancy. She accepted, left to do it but only returned after 4 weeks with complaints of fetal movements and a history of a continuous gush of nonoffensive fluid from the vagina of about 9 days duration in a febrile context. On physical examination, she was anxious, alert, and oriented. Her abdomen was distended with a SFH measuring 18 cm. FHT were present with a fetal heart rate of 140–144 beats/minute. A speculum examination revealed collection of yellowish discharge in the posterior vagina fornix. The cervix was closed, long, posterior, and firm. Two obstetric ultrasounds done 26 days apart (before and after the discharge) were available. The first obstetric ultrasound following initial consultation reported a singleton viable intrauterine pregnancy (IUP) and a subserosa uterine fibroid measuring 3.5 × 2.1 cm; the abdominal ultrasound done just before presentation showed a singleton viable IUP at 25 weeks in breech presentation with severe oligohydramnios. The third obstetric ultrasound repeated in our hospital reported similar findings. A working diagnosis of prolonged premature rupture of membranes (PPROM) complicated by oligohydramnios was made. A full blood count and other biological workups requested and done were normal.

**Fig. 1:**
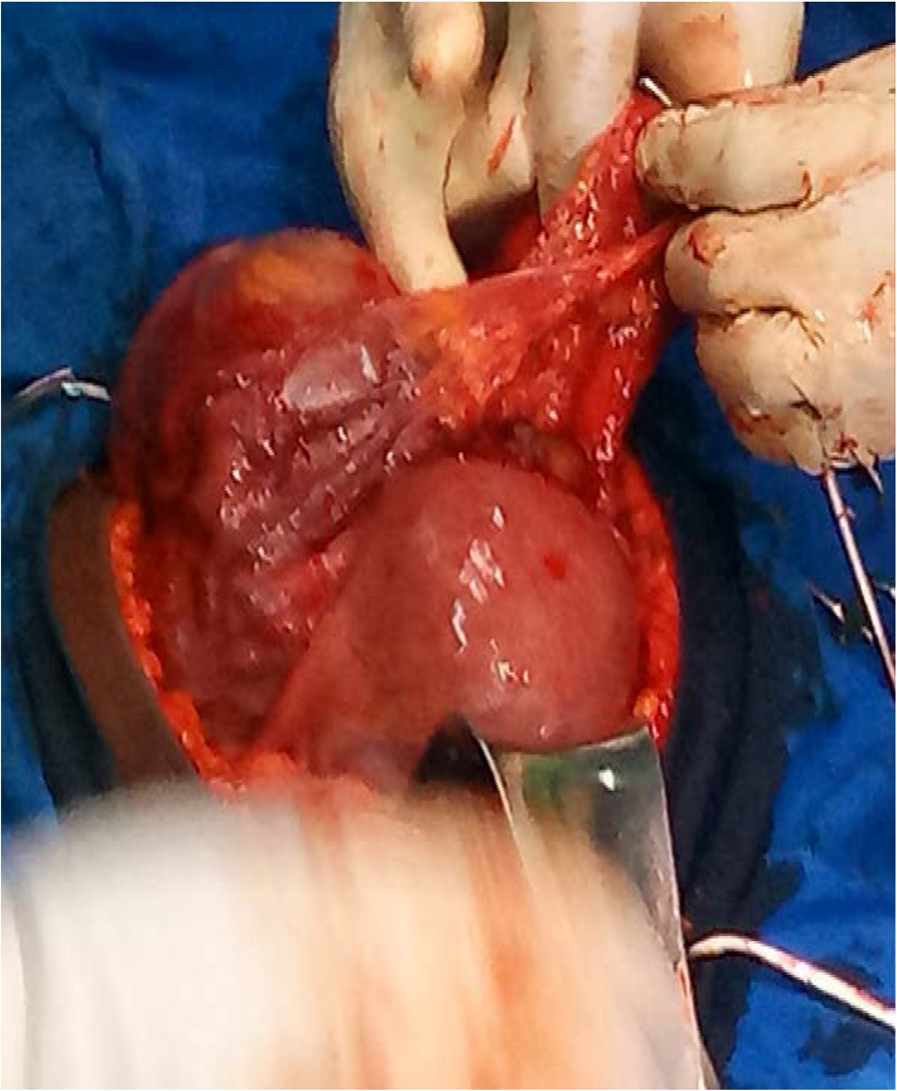
Showing placenta, uterus, and membranes during laparotomy

**Fig. 2:**
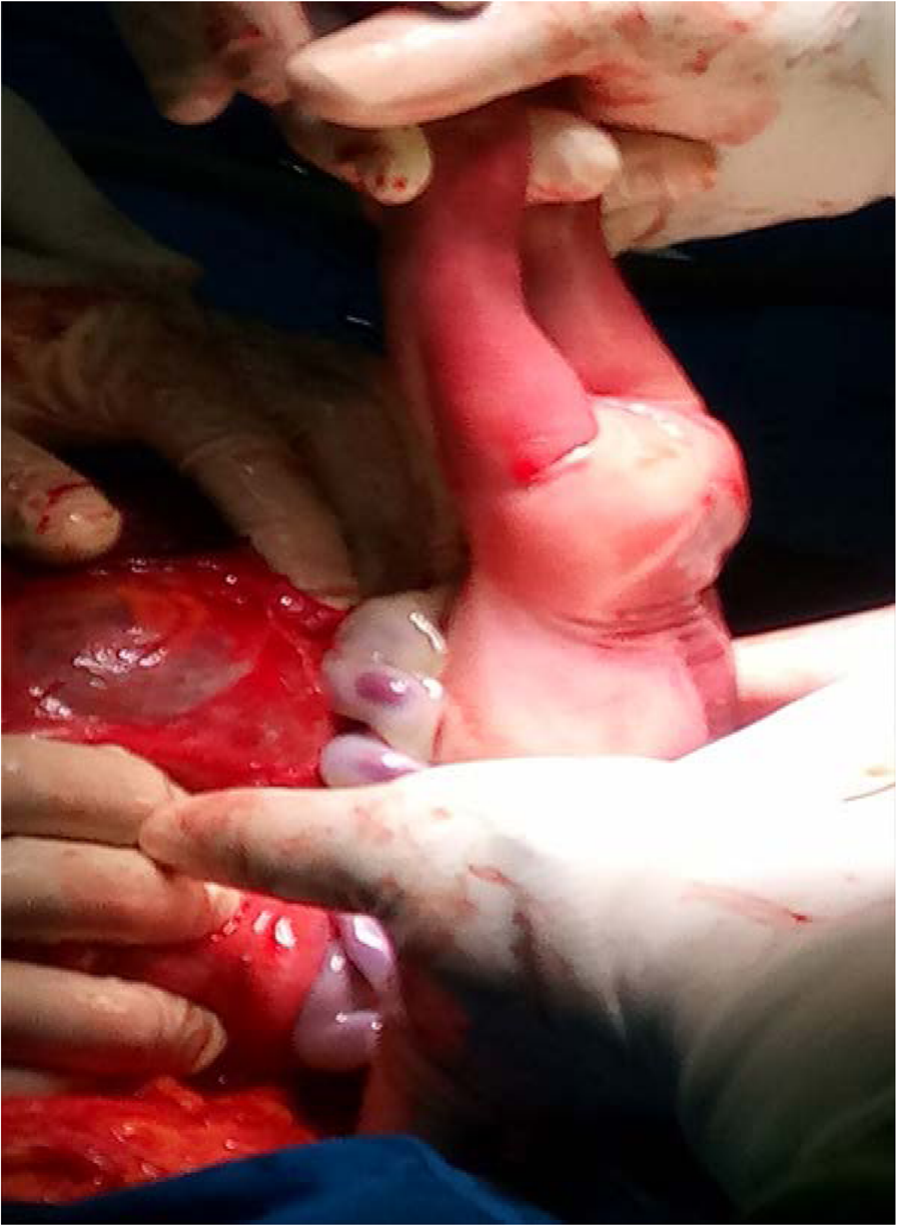
Delivering the baby in breech presentation during laparotomy

She was counseled and she decided to terminate the pregnancy since it was an unwanted pregnancy. She was immediately admitted in the maternity for induction and MTP with misoprostol 50 μg 6 hourly (the dose gradually increased over the days). Other aspects of the treatment included: amoxicillin 1 g 8 hourly, and betamethasone 12 mg 24 hourly for 48 hours. Seventy-two hours later she was revaluated and the Bishop score was persistently poor (3–5/13). With the persistence bogginess of the pouch of Douglas, a fourth obstetric ultrasound was done in our hospital that misdiagnosed the abdominal pregnancy by showing a viable intrauterine pregnancy. An intracervical Foley catheter was then placed to aid in cervical ripening On day 7 of hospitalization, after reevaluation for failed induction and persistent bogginess of the pouch of Douglas, a fifth obstetric ultrasound (transvaginal) was requested. The ultrasound showed a singleton viable intraabdominal pregnancy, placenta attached posteriorly, and the uterus empty. The working diagnosis was changed to an advanced abdominal pregnancy. The patient was counseled on the risks of continuing an AAP and she wanted a termination of the pregnancy since it was previously an unwanted pregnancy. A laparotomy was done on day 8 of hospitalization after diagnosis of the abdominal pregnancy. Intraoperative findings were: bulky uterus with healed fundal perforation, placenta, membranes and viable fetus adhered to the fundus and posteriorly to the large intestine (Fig. 1). A live baby boy was delivered in breech presentation with Apgar scores of 5/10 and 5/10 at first and fifth minute respectively and a weight of 1150 g (Fig. 2). Active bleeding was noted at the cord site that was controlled, her right ovary was edematous, her left ovary and left fallopian tube were not seen and her right fallopian tube and ovary were edematous, tortuous, and fragile. Because the placenta was adherent to the bowel and there was risk of hemorrhage and bowel perforation if removed, the placenta was left *in situ*. On day 1 postlaparotomy, a serum beta human chorionic gonadotrophin (βHCG) test was done and it was raised at 93,864 mIU/mL. Her early postoperative period which consisted of serial monitoring of her abdominal circumference and vital signs was uneventful. She was discharged on day 7 postlaparotomy with a favorable obstetric review and serum βHCG tests to be done twice weekly. Her serum βHCG then declined over the next 3 months to < 5 mIU/mL as shown in Table 1 below. During this follow-up period, counseling was done and she chose intrauterine device (IUD) as the method of contraception, which was inserted 6 weeks following laparotomy.

**Table 1:** Serial serum beta human chorionic gonadotropin postlaparotomy over the 3-month period

Date	Serum βHCG level
05/01/2017	93,864 mIU/mL
19/01/2017	14,260 mIU/mL
02/02/2017	5713 mIU/mL
16/02/2017	1276 mIU/mL
30/02/2017	456 mIU/mL
14/03/2017	127 mIU/mL
30/03/2017	3 mIU/mL

*βHCG* beta human chorionic gonadotropin

## Discussion

Abdominal pregnancy is an extremely rare condition, initially reported in 1708 as an autopsy finding and since then numerous cases have been reported worldwide [3]. Most of the cases of abdominal pregnancies are secondary to an aborted or ruptured tubal pregnancy [10]. Careful assessment suggests that the mechanisms in our indexed case could be primary abdominal pregnancy as the two failed MTP were done late in the first trimester and second trimester reducing the likelihood of secondary abdominal pregnancy from fimbriae abortion and or uterine perforation.

An abdominal pregnancy can go undetected until an advanced gestational age, at which most abdominal pregnancies are discovered, complicating further management [11]. This assertion is true for our case as the pregnancy was not diagnosed until the gestational age was 25 weeks. The clinical symptoms of an uncomplicated abdominal pregnancy described in the literature are very nonspecific, among which the most frequently encountered are persistent abdominal or suprapubic pain (100%), no delay in menstruation, bloody vaginal discharge, gastrointestinal symptoms like nausea and vomiting (70%), painful fetal movements (40%), general malaise (40%), and altered bowel movements [9]. Unfortunately, our patient did not experience many of the above-mentioned symptoms, which could have led at least to a more focused and frequent examination with a chance of discovery; the only symptoms experienced by our patient were persistent fetal movements despite two failed MTP, bogginess in the pouch of Douglas, and oligohydramnios on ultrasound. The most common physical findings reported in the literature are abdominal tenderness (100%), an abnormal fetal lie (70%), easily palpating the baby’s parts on clinical examination, and a displaced uterine cervix (40%) [9]. Most of these signs were absent in our patient. Our patient only had nonspecific abdominal distension and bogginess in the pouch of Douglas on vaginal examinations. Laboratory tests do not have a specific/clear diagnostic value but among the altered ones, were a positive pregnancy test and elevated human chorionic gonadotropin. These tests are more useful in monitoring.

In most of the cases, the diagnosis is usually made by an early obstetric ultrasound. Diagnostic criteria of abdominal pregnancy by an ultrasound may include: demonstration of a fetus in a gestational sac outside the uterus, or the depiction of an abdominal or pelvic mass identifiable as the uterus separate from the fetus; failure to see a uterine wall between the fetus and urinary bladder; recognition of a close approximation of the fetus to the maternal abdominal wall; localization of the placenta outside the confines of the uterine cavity, the classic finding of an empty uterine cavity, which can be associated with no sign of ectopic tubal pregnancy; [12]. Transvaginal ultrasound in our case at 25 weeks revealed some of the suspicious signs mentioned above, which led to the correct diagnosis such as the empty uterine cavity, the gestational sac without any myometrium surrounding it, and oligohydramnios. Our patient had regular transabdominal ultrasounds after failed MTP, all the images revealing a normal pregnancy except the transvaginal ultrasound that was suggestive of abdominal pregnancy. This also highlights the utility of the transvaginal ultrasound in terms of diagnostic ability with respect to abdominal ultrasound. We believe this was due to the addition of the two separate masses, the empty uterus and the gestational sac with a fundal implantation as one due to the dimensional image rendered by transvaginal sonography. As showed in our indexed case above, the incidence of the diagnostic error is very high. Studies have estimated this to be as high as 60% [13], signaling the need for a high index of suspicion and multiple diagnostic procedures in order to lower such a high risk.

AAP is usually associated with very high maternal, fetal and perinatal mortality and morbidity. The maternal mortality rate ranges between 0.5 and 20% [13, 14]. Maternal morbidity and mortality is usually associated to severe hemorrhage, bowel obstruction, perforation, fistula, or disseminated intravascular coagulations [13, 14]. These rates are usually higher if the placenta is left in place as a treatment option [14]. The perinatal mortality classically registers a higher value, of about 40% up until 83–95% [14]. Unfortunately, according to multiple sources, between 21 and 90% of the surviving fetuses have serious birth defects due to compression (lack of the amniotic fluid) and vascular disruption [14]. However, if the neonate survives longer than 1 week; the most frequent complications reported are torticollis, flattening of the head, facial or cranial asymmetry, thoracic malformations, limb defects, joint abnormalities, or central nervous system malformations [9, 14]. In our indexed case here, our patient was counseled on the above complications and immediate termination of pregnancy was decided as the pregnancy was previously unwanted. None of the maternal complications were observed in our patient. The neonate died 3 hours following delivery by laparotomy.

The management of abdominal pregnancy depends on the gestational age at diagnosis and maternal hemodynamic status. As with all types of ectopic pregnancy, medical management of abdominal pregnancy has been reported. Agents used to treat these ectopic pregnancies include methotrexate (systemic and local), local instillation of potassium chloride, hyperosmolar glucose, prostaglandins, danazol, etoposide, and mifepristone. Medical management is usually opted for in early abdominal pregnancy and in pregnancies where surgery may lead to potentially life-threatening bleeding. In AAP and in early abdominal pregnancies with maternal hemodynamic instability, management options included expectant management, laparotomy, and laparoscopy [15, 16]. In our indexed case, considering the risk of pulmonary hypoplasia secondary to severe oligohydramnios and other neonatal morbidities and mortalities described earlier, and lack of local expertise for laparoscopy, surgical management with laparotomy was the option.

Several options have been described for the treatment of the placenta after delivery. This ranges from complete removal through partial removal to leaving the placenta *in situ*. The treatment option depends on the insertion site of placenta [16]. In the case described here, the placenta was left *in situ* as it was deeply adherent to bowels. Leaving the placenta *in situ* with the ligation of the umbilical cord, can be associated with expectant management or other measures, which can accelerate placental trophoblast involution like methotrexate therapy or embolization [13, 14]. According to Kun *et al.* [17], after leaving the placenta *in situ*, the value of βHCG level can regress to a normal value within months [17]. The use of adjuvant methotrexate therapy is still controversial, some considering it helpful in accelerating the placental involution, some disapproving of its use due to the accumulation of necrotic tissue associated with rapid degradation, thus, a higher risk of sepsis. In our patient, the placenta was left *in situ* and expectant management done postoperatively with serial βHCG, which declined to normal over 3 months.

## Conclusions

Abdominal pregnancy is a rare form of ectopic pregnancy with very high morbidity and mortality for both the mother and the fetus at advanced gestation. Whenever there is failed MTP at any gestation, experienced radiologists should try to confirm the site of the pregnancy at earlier gestation so that medical management can be given earlier to reduce blood flow and subsequent complications associated with laparotomy.

Even in the era of increased access to advanced diagnostic imaging modalities, the diagnosis and management of AAP is still a challenge to obstetricians. Albeit the relative rarity of advanced abdominal pregnancy in sub-Saharan Africa, this case highlights the importance of thorough early clinical assessment and comprehensive ultrasound assessment of patients with presumptive symptoms of pregnancy. Furthermore, limited resources akin to low-income settings contribute to the health burden associated with intraabdominal pregnancy. High index of suspicion and timely surgical intervention is imperative to decrease maternal and fetal complications. Obstetricians and radiologists should improve their skills to diagnose these cases in time, so that they do not reach an advanced stage where management itself becomes difficult.

## Abbreviations

AAP: Advanced abdominal pregnancy
βHCG: beta human chorionic gonadotropin
MTP: Medical termination of pregnancy
SFH: Symphysiofundal height

## References

[1] Okafor I, Ude A, Aderibigbe A, Amu O, Udeh P, Obianyo N, *et al.* Abdominal pregnancy- a case report. J West Afr Coll Surg. 2011;1(1):121–30. PMC4170250 25452947

[2] Worley KC, Hnat MD, Cunningham FG. Advanced extrauterine pregnancy: diagnostic and therapeutic challenges. Am J Obstet Gynecol. 2008;198(3):e1–297. e7. 10.1016/j.ajog.2007.09.044 18313451

[3] Baffoe P, Fofie C, Gandau BN. Term abdominal pregnancy with healthy newborn: a case report. Ghana Med J. 2011;45(2):81–3. 10.4314/gmj.v45i2.68933 PMC3158531 21857726

[4] El-Agwany AS, El-badawy E, El-habashy A, El-gammal H, Abdelnaby M. Secondary abdominal pregnancy after suspected ruptured cornual pregnancy with good maternal outcome: a case with unusual gangrenous fetal toes and ultrasound diagnoses managed by hysterectomy. Clin Med Insights Womens Heal. 2016;9:1–5. 10.4137/CMWH.S36311 PMC4743745 26862301

[5] Gupta P, Sehgal A, Huria A, Mehra R. Secondary abdominal pregnancy and its associated diagnostic and operative dilemma: three case reports. J Med Case Rep. 2009;3:7382. 10.4076/1752-1947-3-7382 PMC2737786 19830195

[6] Cunningham FG. Williams obstetrics. 24th ed. New York: McGraw-Hill Medical; 2014. p. 1358.

[7] Huang K, Song L, Wang L, Gao Z, Meng Y, Lu Y. Advanced abdominal pregnancy: an increasingly challenging clinical concern for obstetricians. Int J Clin Exp Pathol. 2014;7(9):5461–72. PMC4203159 25337188

[8] Dahab AA, Aburass R, Shawkat W, Babgi R, Essa O, Mujallid RH. Full-term extrauterine abdominal pregnancy: a case report. J Med Case Reports. 2011;5:531. 10.1186/1752-1947-5-531 PMC3216095 22040324

[9] Bohiltea R, Radoi V, Tufan C, Horhoianu I, Bohiltea C. Abdominal pregnancy - Case presentation. J Med Life. 2015;8(1):49–54. PMC4397520 25914739

[10] Amritha B, Sumangali T, Priya B, Deepak S, Sharadha R. A rare case of term viable secondary abdominal pregnancy following rupture of a rudimentary horn: a case report. J Med Case Rep. 2009;3:38. 10.1186/1752-1947-3-38 PMC2640409 19178737

[11] Molinaro TA, Barnhart KT. Ectopic pregnancies in unusual locations. Semin Reprod Med. 2007;25(2):123–30. 10.1055/s-2007-970051 17377899

[12] Allibone GW, Fagan CJ, Porter SC. The sonographic features of intra-abdominal pregnancy. J Clin Ultrasound. 1981;9(7):383–7. 10.1002/jcu.1870090706 6792237

[13] Rahman MS, Al-Suleiman SA, Rahman J, Al-Sibai MH. Advanced abdominal pregnancy--observations in 10 cases. Obstet Gynecol. 1982;59(3):366–72. 7078883

[14] Nkusu Nunyalulendho D, Einterz EM. Advanced abdominal pregnancy: case report and review of 163 cases reported since 1946. Rural Remote Health. 2008;8(4):1087. 19053177

[15] Agarwal N, Odejinmi F. Early abdominal ectopic pregnancy: challenges, update and review of current management. Obstet Gynaecol. 2014;16(3):193–8.

[16] Zinger M, Rosenfeld D. Failed treatment of abdominal pregnancy with methotrexate. A case report. J Reprod Med. 2001;46(4):392–4. 11354843

[17] Kun KY, Wong PY, Ho MW, Tai CM, Ng TK. Abdominal pregnancy presenting as a missed abortion at 16 weeks’ gestation. Hong Kong Med J Xianggang Yi Xue Za Zhi. 2000;6(4):425–7. 11177167

